# Dr. Williams’s Retort

**Published:** 1896-11

**Authors:** 


### DR. WILLIAMS'S RETORT.

In the October number of the *Dental Cosmos*, Dr. J. Leon Williams, of London, devotes three pages of solid matter to the editor of this journal. The fact that so much time and space has been taken must be regarded as complimentary, although, in our opinion, it might have been used for a better purpose.

That the editor of the *Dental Cosmos* does not agree with this view is apparent, for he takes occasion to say, by way of preface, that "in the interest of justice and right, matters of this character are entitled to immediate publication." We are pleased to learn, and, doubtless, the dental profession will be equally delighted to note, that the editor has adopted a broad and liberal policy for the *Dental Cosmos*, for he says, alluding to the article of Dr. Williams, "It is merely an expression of the general policy of the journal to afford opportunity for a fair hearing of all sides of a question of this character." In fact, the editor of the *Dental Cosmos* was so much impressed with its importance that he adopted the unusual course of placing it upon the editorial pages, and states that this was done "because it was received after that part of the *Dental Cosmos* devoted to 'Correspondence' had been printed." The curious reader on turning over the pages will look in vain for the part "devoted to correspondence."

It is quite evident that Dr. Williams found a club to use on Heintzmann, Bödecker, and Abbott, when he originally assumed to criticise the National Association of Dental Faculties, and now he proposes to himself to make of the editor of this journal a similar club, and for the same purpose. We do not intend to be thus used.

In the first place, we can state to Dr. Williams that this journal does not constitute itself the defender of the action of the Association of Faculties, nor the trinity of investigators mentioned. The editor would not arrogate to himself the position of a court to decide disputed questions in histology, and while he is personally not in full accord with the histological views held by Bödecker, Heintzmann, and Abbott, he would deem himself unworthy of a place in a liberal profession if he refused to give to these views, and all others, due consideration, nor would he lower his self-respect by descending to the murky region of abuse.

When Dr. Williams asserts that the editorial in the International Dental Journal meant to convey the impression that the Association of Faculties was above criticism he distorts the truth. He changes the real statement made in his first paragraph, and adds an additional line in which he seeks to impute motives not held, and then in the second paragraph he states that the editor of the Journal "is not speaking for the entire board of the National Association of Dental Faculties *when he intimates that they are above and beyond criticism*." (Italics ours.) At no time has the editor ever sought to speak for that association, but what he did say, August, 1896, was, "The position held by the National Association of Dental Faculties is a dignified and proper one, and is not likely to be changed by any criticism, come from what quarter it may."

Here is another of his statements: "Now, what are the facts about this matter. It seems to me that the editor of the INTERNATIONAL DENTAL JOURNAL has done his best to conceal by a *most equivocal* and disingenuous statement." Conceal what? It is a new sensation to the writer to be accused of equivocation, the twin sister of lying. The statement we made was an open one as an editor and as a member of the Association of Faculties. We stated in the editorial that "the Association of Faculties had nothing directly to do with the teachings of Dr. Williams, Dr. Bödecker, or Dr. Abbott, but leaves the matter to the judgment of its individual members." That may be equivocal, but if so, the word has a different meaning in England from that usually given it in America.

Here is another of his reliable quotations: "The next statement which the editor of the INTERNATIONAL DENTAL JOURNAL would, if accepted, place the board [what board?] in a still more unenviable position. The indorsement, he says, simply means that an author has published a book *worthy of men engaged in teaching*." (Italics ours.) What the editorial in question did say was this: "The recommendation [of the Association of Faculties] does not go so far as to even suggest that any work so recommended shall be used as a text-book in the colleges, although that word has been used, but it simply means that an author has published a book worthy of *consideration* by men engaged in teaching, and does not carry any force beyond that statement." His leaving out the word *consideration*, to say nothing of the explanatory matter, leads to a marked difference in the sense of the sentence. This may have been an unintentional omission, and we are disposed to give Dr. Williams the benefit of the doubt.

We shall make one more quotation and then rest, for it would be a useless expenditure of strength to follow his methods of distorting statements: "Dr. Bödecker says the National Association of Dental Faculties has adopted his book as a text-book for students. His statement has not been officially denied, but the editor of the INTERNATIONAL DENTAL JOURNAL takes it upon himself to say that it has not only *not* been adopted, but that the board has not even suggested that it be so used." We do not choose to make use of the proper word to characterize this, but refer our readers to the editorial, where not a word will be found to give color to such a statement.

We have not the space or the disposition to follow Dr. Williams through his long arraignment. We are not particularly disturbed by it, but it may be suggested that in our opinion it is not in good taste for a dweller on English soil, for all the good that may be there ingathered, to criticise an association with which he can have but a remote interest. If he chooses to attack Bödecker's book from his foreign stand-point he should do it fairly or omit it altogether. This journal is not the apologist of any author or of any aggregation of men, but if anything is to be said we propose to use words without reserve, and which will be understood. It is not its purpose either to permit men to be called fools by quoting a possibly mythical "eminent teacher in Germany," who is made to say "Messrs. Heintzmann and Bödecker have made immortal fools of themselves in several treatises."

The writer of this makes no claim to a knowledge of general or special histology equal to that possessed by Dr. Williams, but the little that has been absorbed was sufficient to interest him in every line of Dr. Williams's recent work, and to appreciate it as it deserves, as a most valuable, if not the most valuable contribution made upon the subjects treated in the past decade. This, however, does not mean indorsement of all his views. With this liberal spirit all things should be met, and any other course is to the intelligent reasoner unprofessional.

We leave Dr. Williams here, and do not expect to recur to the subject again. He can fight his windmills through the pages of the *Dental Cosmos*, if so disposed, but we have no disposition to take part in any such controversy.

